# Exploring the carcinogenic potential of bisphenol A in lung adenocarcinoma: molecular mechanisms, key gene insights, and immune microenvironment impacts

**DOI:** 10.3389/fimmu.2025.1647807

**Published:** 2025-10-16

**Authors:** Haizhu Chen, Tiancheng Jiang, Yihan Yang, Gengyi Cai, Yupeng Jiang, Wenhao Ouyang

**Affiliations:** ^1^ Guangdong Provincial Key Laboratory of Malignant Tumor Epigenetics and Gene Regulation, Breast Tumor Centre, Sun Yat-sen Memorial Hospital, Sun Yat-sen University, Guangzhou, China; ^2^ School of Medicine, Sun Yat-sen University, Shenzhen, China; ^3^ Jiangxi Provincial Key Laboratory of Respiratory Diseases, Jiangxi Institute of Respiratory Diseases, Department of Respiratory and Critical Care Medicine, The First Affiliated Hospital, Jiangxi Medical College, Nanchang University, Nanchang, China; ^4^ Department of Oncology, The Second Xiangya Hospital, Central South University, Changsha, China; ^5^ Department of Medical Oncology, Sun Yat-sen Memorial Hospital, Sun Yat-sen University, Guangzhou, China

**Keywords:** bisphenol A, lung adenocarcinoma, oncogenic genes, tumor microenvironment, molecular docking

## Abstract

**Background:**

Bisphenol A (BPA) is an endocrine-disrupting chemical that may contribute to cancer development. However, its role in lung adenocarcinoma (LUAD) remains poorly understood. This study aimed to investigate how BPA affects LUAD development by examining key genes involved in tumor progression and the immune microenvironment.

**Methods:**

Network toxicology, molecular docking, and clinical data analyses were performed to identify potential molecular targets of BPA in LUAD. Common genes between BPA targets and LUAD biomarkers were screened, and their biological significance was evaluated through survival analysis, immune infiltration assessment, and protein–ligand interaction studies.

**Results:**

A total of 218 overlapping genes were identified between BPA targets and LUAD biomarkers, including BUB1, BUB1B, CCNA2, CDK1, and UBE2C. These genes were strongly associated with LUAD progression, poor survival outcomes, and enhanced immune cell infiltration. Molecular docking revealed strong binding affinities between BPA and these proteins, suggesting potential disruption of their normal biological functions.

**Conclusion:**

This study provides valuable insights into the potential risks of BPA exposure in LUAD. The identified key genes and pathways may serve as potential biomarkers and therapeutic targets, offering new directions for future research and public health strategies.

## Introduction

Bisphenol A (BPA) is a synthetic compound widely used in plastics, food packaging, and epoxy resins (1). As an endocrine disruptor, BPA can mimic or interfere with hormone signaling, thereby affecting cell proliferation, apoptosis, and gene expression. Previous studies have linked BPA exposure to cancers such as breast, prostate, and live (2).

However, its potential impact on lung cancer, particularly lung adenocarcinoma (LUAD), remains poorly understood. LUAD is one of the most common subtypes of non–small cell lung cancer, with incidence rising globally (3, 4). While smoking, air pollution, and occupational carcinogen exposure are established risk factors (5, 6), the contribution of endocrine-disrupting chemicals such as BPA has received limited attention. Preliminary evidence suggests that BPA may influence lung epithelial cells through oxidative stress, inflammation, or estrogen receptor–mediated pathways (7–9), but direct mechanistic insights in LUAD are still lacking. This gap highlights the importance of clarifying whether and how BPA contributes to LUAD initiation and progression, which could inform both risk assessment and public health policy.

This study integrates network toxicology, molecular docking, and clinical data analysis to investigate how BPA contributes to LUAD. Network toxicology identifies key pathways and target genes, molecular docking predicts BPA–protein interactions, and clinical data validate their relevance. Together, these approaches provide a mechanistic basis for BPA’s carcinogenic potential in LUAD and support future risk assessment and prevention strategies.

## Methods

### Biotoxicity prediction for BPA-induced toxicity assessment

The integration of network search algorithms and biotoxicity prediction methods within specialized software tools enables us to utilize structured models to predict the toxicity associated with BPA compounds. By employing a total of three software tools, including ADMETlab 2.0 and ADMET as initial screening platforms, our goal is to obtain fundamental yet accurate insights into BPA-induced toxicity (10, 11). Subsequently, the potential molecular targets of BPA were retrieved from the ChEMBL database, restricted to Homo sapiens, and standardized to official gene symbols for downstream analysis (12).

### Identifying biomarkers for LUAD

The RNA (mRNA) expression dataset used in this study for LUAD analysis was obtained from The Cancer Genome Atlas (TCGA) database (https://portal.gdc.cancer.gov/). This dataset includes 59 normal samples and 535 tumor tissue samples, enabling the identification of differentially expressed genes (DEGs) considered as LUAD-specific biomarkers (13). To explore the potential mechanisms of BPA-induced LUAD, the identified DEGs were intersected with BPA target genes, allowing the determination of potential BPA-related targets involved in LUAD progression.

### Protein-protein interaction network analysis

The STRING database was utilized to perform PPI network analysis for potential biomarkers linking BPA and LUAD. The “multiple proteins” option was selected, with the species specified as Homo sapiens. Interactions with a *P* < 0.05 were considered statistically significant, and an interaction score > 0.7 was regarded as a high-confidence relationship. Cytoscape software (version 3.6.0) was employed to visualize the PPI network, while the CytoHubba plugin was used to identify the top five hub genes within the network.

### Functional and pathway enrichment analysis

The targeted gene was analyzed for Gene Ontology (GO) and Kyoto Encyclopedia of Genes and Genomes (KEGG) enrichment using the R package “clusterProfiler”. GO annotations were divided into three main categories: biological processes (BP), cellular compartments (CC), and molecular functions (MF). Terms in GO and KEGG with an adjusted P-value below 0.05 were considered enriched. Visualization of these terms was achieved using the R packages “enrichplot” and “ggplot2”. Furthermore, to investigate gene–pathway associations, we employed the Metascape database (https://metascape.org/gp/index.html#/main/step1). This platform integrates information from more than 40 resources and groups genes into clusters when terms meet the thresholds of *P* < 0.01, at least three associated genes, and an enrichment factor > 1.5. This approach facilitates the discovery of significantly enriched pathways and functional annotations related to hub genes.

### Molecular docking of BPA with core target proteins in LUAD

Molecular docking was utilized to predict the optimal binding orientation of bisphenol A (BPA; PubChem CID: 6623) with selected target proteins, providing structural insights into potential interaction mechanisms. A semi-flexible docking approach was employed to enable the formation of stable ligand–receptor complexes, which is fundamental in structure-guided drug discovery and in screening candidate compounds.

The three-dimensional structure of BPA was retrieved from the PubChem database and subjected to model construction and energy minimization in ChemDraw 20.0. Protein structures for BUB1 (UniProt ID: O43683), BUB1B (O60566), CCNA2 (P20248), CDK1 (P06493), and UBE2C (O00762) were obtained from the AlphaFold Protein Structure Database. Prior to docking, proteins were processed in PyMOL 2.4 by deleting crystallographic water molecules and non-essential ligands, followed by hydrogen atom addition. The processed structures were converted into PDBQT format using AutoDock Tools 1.5.6.

Docking simulations were performed with AutoDock Vina 1.1.2, with the grid box adjusted to encompass the entire protein structure while maintaining default settings for other parameters. For each ligand–protein pair, the top ten binding conformations were generated, and the pose with the lowest binding energy and highest cluster occurrence was selected as the most plausible interaction model. All simulations were run in triplicate, and binding energies were expressed as mean ± standard deviation. Visualization and interaction analysis of the resulting complexes were carried out using PyMOL 2.4 and Discovery Studio 2019 (14).

### Overall survival analysis

To evaluate the prognosis of LUAD, survival follow-up data for each patient were extracted from the TCGA-LUAD dataset. Patients were divided into two groups based on the optimal cutoff value for each gene’s expression level. Kaplan-Meier (KM) survival curves were used to assess the association between gene expression and patient survival. Among the five hub genes analyzed, significant correlations were identified, with statistical significance determined using the log-rank test.

### Estimation of immune cell infiltration

The immune cell infiltration levels within the tumor microenvironment (TME) of each LUAD patient were estimated using the CIBERSORT algorithm, which identifies RNA transcriptional profiles of distinct cell subtypes. The “CIBERSORT” R package was employed to evaluate the relative abundance of 22 tumor-infiltrating immune cell types in LUAD samples, including naive B cells, memory B cells, CD8^+^ T cells, naive CD4^+^ T cells, resting/activated memory CD4^+^ T cells, regulatory T cells (Tregs), follicular helper T cells, γδ T cells, resting/activated dendritic cells, M0/M1/M2 macrophages, eosinophils, neutrophils, resting/activated mast cells, resting/activated NK cells, monocytes, and plasma cells, P values < 0.05 were considered statistically reliable and retained for downstream analyses.

### Single-cell RNA sequencing analysis

In this research, single-cell RNA sequencing (scRNA-seq) data were obtained from the GSE143423 dataset available through the TISCH database (15). This dataset was used to pinpoint and describe distinct cell subsets present in LUAD samples. All datasets in TISCH undergo a unified pipeline including quality control filtering, normalization, dimensionality reduction, clustering, and cell type annotation based on canonical marker genes from the original studies. Batch effect correction is implemented through the Seurat integration pipeline, which uses canonical correlation analysis (CCA) and mutual nearest neighbor (MNN)–based anchoring to align data across batches (16). Through this analysis, specific cell populations expressing critical biomarkers were identified.

### Cell culture

The immortalized human bronchial epithelial cell line BEAS-2B was obtained from the American Type Culture Collection (ATCC, Manassas, VA, USA). Cells were thawed and expanded according to ATCC protocols. BEAS-2B cells were cultured in Dulbecco’s Modified Eagle Medium (DMEM) supplemented with 10% fetal bovine serum (FBS; Gibco, USA) and 1% penicillin-streptomycin (Invitrogen, USA), and maintained at 37°C in a humidified incubator with 5% CO_2_ atmosphere.

### Cell viability assay

BEAS-2B cells were seeded into 96-well plates (5000 cells/well). After overnight incubation, cells were treated with various concentrations of BPA (0–2560 nM) for 48 hours. Cell viability was determined using the CCK-8 assay according to the manufacturer’s instructions. Absorbance was measured at 450 nm using a microplate reader.

### Colony formation assay

Cells were seeded into 6-well plates (1000 cells/well) and treated with indicated BPA concentrations for 14 days. Colonies were fixed with methanol, stained with 0.1% crystal violet, and colonies containing more than 50 cells were counted under a microscope.

### Quantitative real-time PCR

Total RNA was isolated using TRIzol reagent (Vazyme Biotech Co., Ltd., Nanjing, China) following the manufacturer’s protocol. One microgram of RNA was then reverse transcribed into cDNA with the HiScript^®^ III First Strand cDNA Synthesis Kit (Vazyme Biotech Co., Ltd., Nanjing, China). Quantitative PCR was conducted on a QuantStudio™ Dx Real-Time PCR System (Applied Biosystems, MA, USA) employing the ChamQ Universal SYBR^®^ qPCR Master Mix (Vazyme Biotech Co., Ltd., Nanjing, China). The cycling program consisted of an initial activation at 95°C for 30 s, followed by 35 cycles of denaturation at 95°C for 5 s and annealing/extension at 60°C for 30 s. Relative gene expression was determined using the 2^–ΔΔCt^ approach, with GAPDH as the internal reference. The corresponding primer sequences are provided (**Table 1**).

**Table 1:** Primer sequences used in this study.

Gene	Forward primer (5’-3’)	Reverse primer (5’-3’)
BUB1	AGGAAAGATGTGGCTGAGGA	TGAGAGCTGCTGAGGAAGGT
BUB1B	TTGTGGAGCTGCTGTTTGGG	GCAGGACTTAGTGTCTGCCC
CCNA2	AGTCCAGGAAGAGGAAGTGC	CTTGAGCTTGTCTCCAGGTC
UBE2C	CGACTGCTGGGTATCCTTGA	AGGGACAGTCCATGATGCAC
CDK1	AAGGAAGATGGCCGATGAGA	CCAGGGATGATTCAGTGCCAT
GAPDH	AATCCCATCACCATCTTCCA	TGGACTCCACGACGTACTCA

### Western blotting

Cells were lysed in RIPA buffer (Beyotime, Shanghai, China) supplemented with protease inhibitor cocktail (Beyotime, Shanghai, China), and total protein concentrations were determined using a BCA Protein Assay Kit (Beyotime, Shanghai, China). Equal amounts (30 μg) of protein were separated on 10% SDS–PAGE gels and transferred to PVDF membranes (Millipore, Billerica, MA, USA). Membranes were blocked with 5% non-fat milk in TBST for 1 h at room temperature, followed by overnight incubation at 4 °C with the following primary antibodies: anti-BUB1 (1:1000, Proteintech, Cat# 13330-1-AP), anti-BUB1B (1:1000, Proteintech, Cat# 11504-2-AP), anti-CCNA2 (1:1000, Proteintech, Cat# 18202-1-AP), anti-UBE2C (1:1000, Proteintech, Cat# 66087-1-Ig), anti-CDK1 (1:1000, Proteintech, Cat# 10762-1-AP), and anti-GAPDH (1:2000, Proteintech, Cat# 81640-5-RR). After washing, membranes were incubated with HRP-conjugated secondary antibodies (1:5000, Beyotime, Shanghai, China) for 1 h at room temperature. Protein bands were visualized using an enhanced chemiluminescence kit (Thermo Fisher Scientific, USA) and imaged on a ChemiDoc™ XRS+ system (Bio-Rad, Hercules, CA, USA).

## Results

### Identification of biomarkers for LUAD

**Figure 1:**
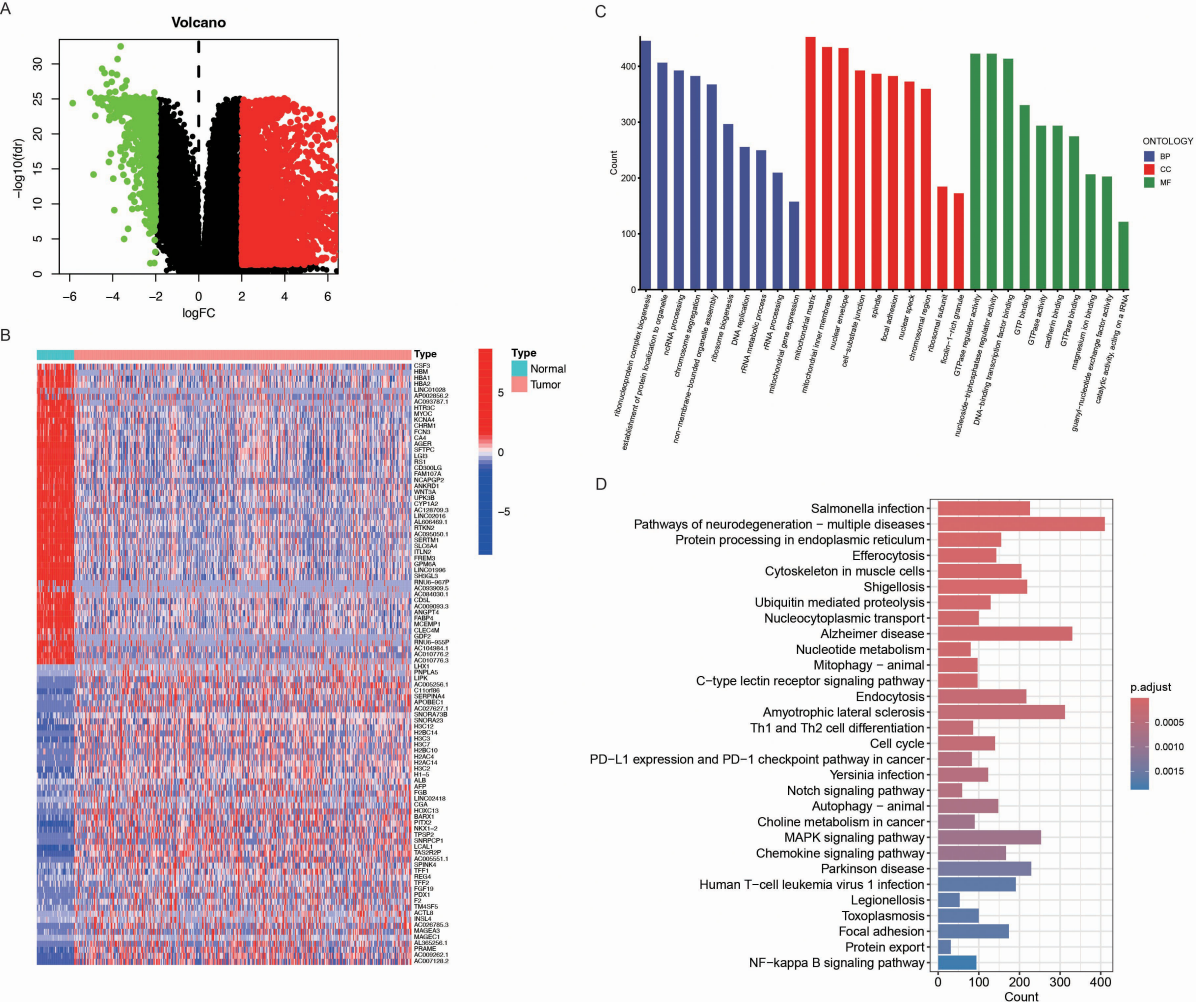
Identification of differentially expressed genes (DEGs) in LUAD. **(A)** Volcano plot of DEGs between LUAD tumor tissues and normal lung tissues. Upregulated genes (logFC > 2, p < 0.05) are shown in red, downregulated genes (logFC < -2, p < 0.05) in green, and non-significant genes in black. **(B)** Hierarchical clustering heatmap of the top DEGs in LUAD and normal samples. The color scale represents the expression level (red: high expression, blue: low expression), and clear clustering patterns distinguish tumor from normal tissues. **(C)** Gene Ontology (GO) enrichment analysis of DEGs, categorized into biological processes (BP, blue), cellular components (CC, red), and molecular functions (MF, green). The x-axis represents GO terms, while the y-axis indicates the gene count associated with each term. **(D)** Kyoto Encyclopedia of Genes and Genomes (KEGG) pathway enrichment analysis of DEGs. The bar plot displays enriched pathways, with the x-axis indicating the number of genes involved in each pathway. The color gradient represents the adjusted p-value, with darker shades indicating higher significance.

First, we performed a differential expression analysis comparing tumor and normal lung tissues. Differentially expressed genes (DEGs) were selected based on a log fold change (logFC) greater than 2 or less than -2, and a p-value less than 0.05. This approach identified a total of 3463 differential expressed genes in LUAD (**Figure 1A**). To further explore the gene expression patterns, we constructed a hierarchical clustering heatmap (**Figure 1B**), which clearly separated LUAD from normal lung tissues. The distinct clustering of the tumor and normal samples further emphasized the unique molecular signature of LUAD and supported the identification of DEGs as potential biomarkers. Additionally, Gene Ontology (GO) enrichment analysis (**Figure 1C**) revealed significant enrichment of DEGs in biological processes such as immune response, cell cycle regulation, and DNA damage repair, which are known to be critical for tumorigenesis. The enrichment in molecular functions like GTPase regulator activity and nucleoside-triphosphatase regulator activity further highlighted the importance of these genes in regulating cell signaling and transcription. In cellular components, DEGs were enriched in regions such as the nuclear envelope and mitochondrial matrix, indicating their roles in cellular structure and tumor metastasis. Moreover, KEGG pathway analysis (**Figure 1D**) highlighted key cancer-related pathways, including the p53 signaling pathway, apoptosis, and immune response pathways, underscoring their relevance in LUAD progression.

### Identification of targets in BPA-induced LUAD progression

**Figure 2:**
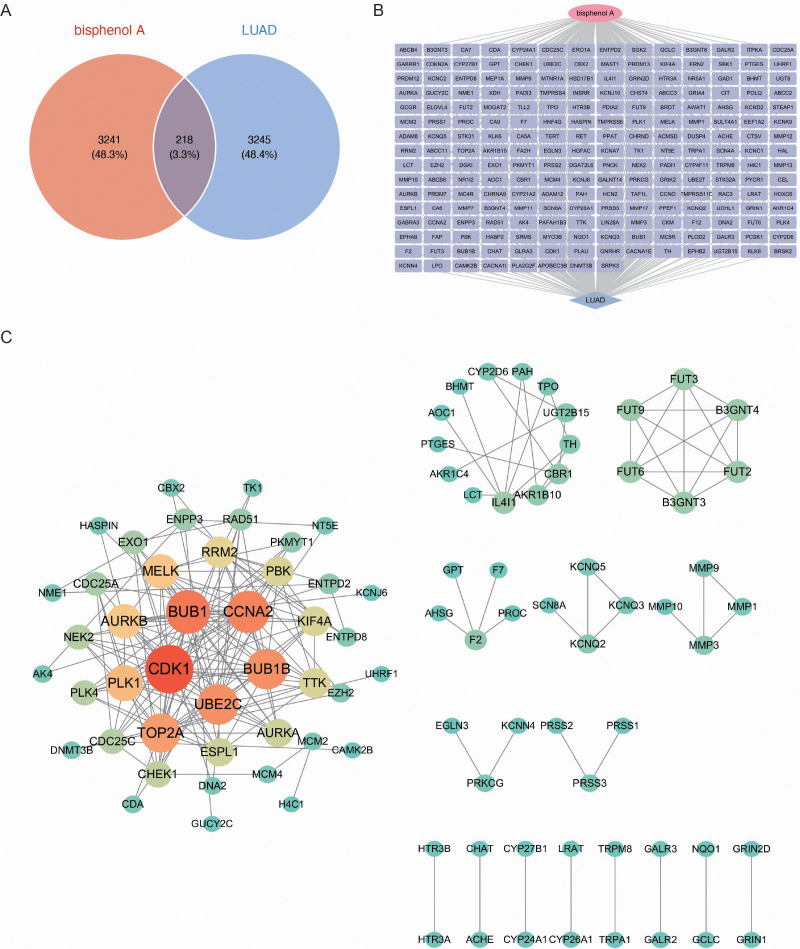
Identification and network analysis of common genes between BPA targets and LUAD biomarkers. **(A)** Venn diagram showing the intersection of genes associated with BPA and LUAD biomarkers. A total of 218 common genes (3.3% of both datasets) were identified, which may play a pivotal role in the effects of BPA on LUAD progression. **(B)** Gene interaction network of the 218 common genes between BPA and LUAD. **(C)** Protein-Protein Interaction (PPI) network showing the relationships between the common genes. Key hub genes such as CDK1, BUB1, CCNA2, UBE2C, and BUB1B are highlighted in red and orange, indicating their central roles in the network.

We performed an intersection analysis of BPA potential targets and LUAD biomarkers to identify common genes. The analysis revealed 218 common genes, which account for 3.3% of both datasets (**Figure 2A**). These common genes may play a pivotal role in mediating the effects of BPA on LUAD progression, making them critical candidates for further study as potential biomarkers.

Next, a network was constructed for the 218 common genes to visualize the interactions between them (**Figure 2B**). This network analysis highlighted the intricate relationships among the genes, indicating their possible involvement in shared biological pathways that contribute to both BPA toxicity and LUAD development. To further explore the functional relationships among these genes, a Protein-Protein Interaction (PPI) network was built (**Figure 2C**). The PPI network revealed several key hub genes, including CDK1, BUB1, CCNA2, UBE2C, and BUB1B, which were highly interconnected. This close network connectivity indicates that these genes’ functions are tightly coordinated within regulatory pathways, indicating their central roles in both BPA-induced toxicity and LUAD progression.

### Functional analysis of targets and pathway enrichment evaluation

**Figure 3:**
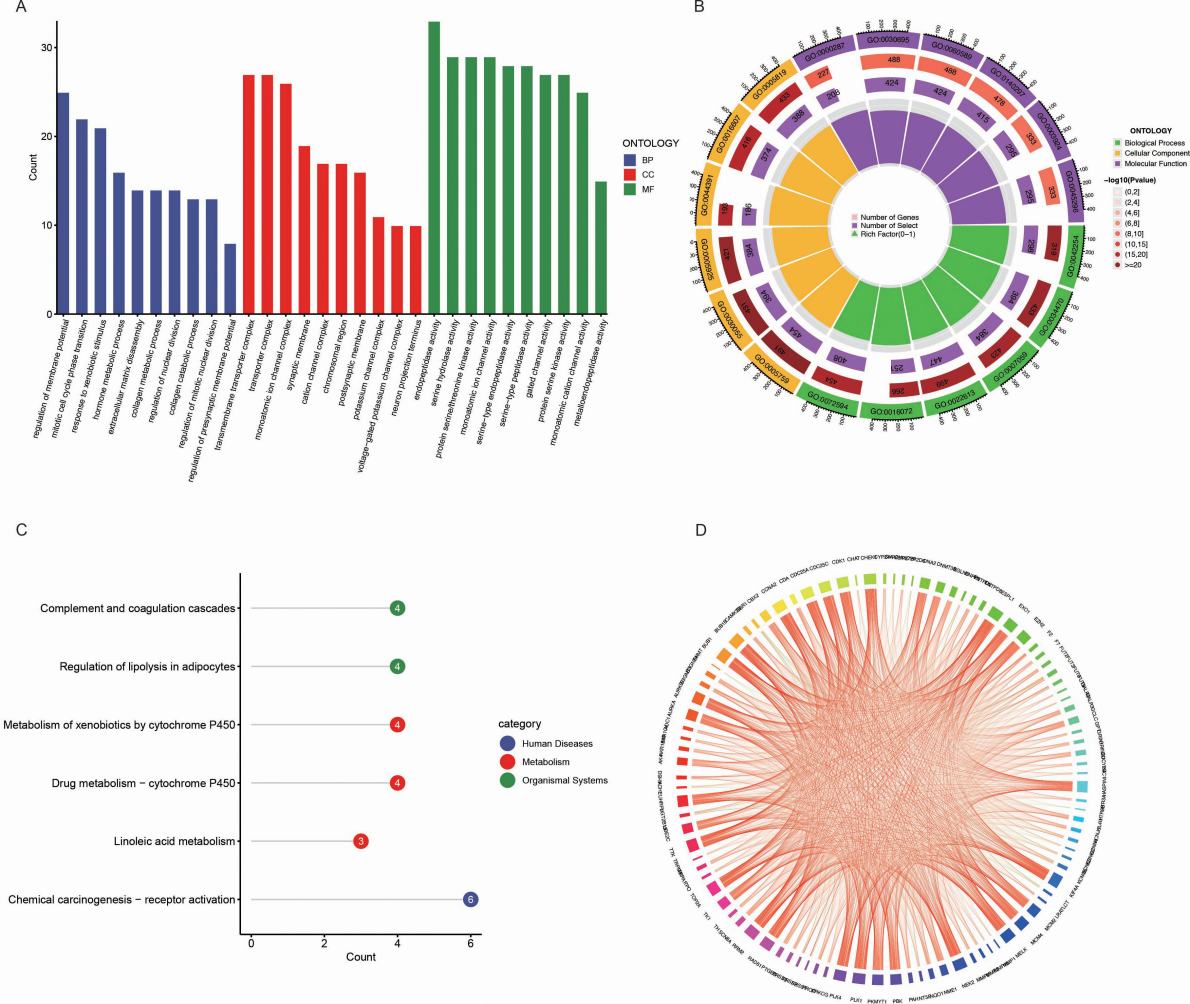
Functional and pathway enrichment analysis of common genes between BPA targets and LUAD biomarkers. **(A)** Gene Ontology (GO) enrichment analysis of the common genes between BPA targets and LUAD biomarkers. The genes are categorized into biological processes (BP, blue), cellular components (CC, red), and molecular functions (MF, green). **(B)** Circular visualization of GO enrichment analysis showing the number of genes associated with each GO term. The inner rings represent various GO categories, and the outer ring color represents the significance level of each term, with darker shades indicating more significant enrichment. **(C)** KEGG pathway enrichment analysis of the common genes, highlighting pathways such as “Complement and coagulation cascades,” “Regulation of lipolysis in adipocytes,” and “Chemical carcinogenesis - receptor activation” (blue). **(D)** Pathway interaction network illustrating the relationships between enriched pathways and genes.

We performed Gene Ontology (GO) enrichment analysis on the 218 common genes identified between BPA potential targets and LUAD biomarkers. The GO analysis revealed significant enrichment in biological processes, including immune response regulation, cell cycle regulation, and metabolic processes, all of which are known to play a crucial role in tumorigenesis and cancer progression (**Figure 3A**). In cellular components, the genes were predominantly enriched in areas such as the plasma membrane and cytoplasm, indicating their involvement in intercellular signaling and cellular communication. Molecular functions related to protein binding, GTPase activity, and enzyme regulation were also enriched, further revealing their key roles in cellular signaling and regulation of various metabolic pathways. These results highlight the potential functional roles of these genes in both BPA exposure and LUAD development.

Further visualization of the GO enrichment results using a circular plot demonstrated the number of genes associated with each GO term, color-coded by significance (**Figure 3B**). Biological processes such as RNA metabolism and protein localization were the most enriched, emphasizing their importance in cellular function and stability. In the molecular function category, GTPase activity and DNA-binding transcription factor activity were highly enriched, indicating these genes’ involvement in gene expression regulation and cellular signaling.

In the KEGG pathway enrichment analysis, several pathways were found to be enriched, including “Complement and coagulation cascades,” “Regulation of lipolysis in adipocytes,” and “Metabolism of xenobiotics by cytochrome P450” (**Figures 3C, D**). These pathways are critical for processes involving immune responses, lipid metabolism, and detoxification, which are potentially influenced by both BPA exposure and LUAD progression. Additionally, the “Chemical carcinogenesis – receptor activation” pathway was enriched, further linking BPA’s carcinogenic effects to LUAD development.

Metascape analysis demonstrated that BPA-induced lung adenocarcinoma is associated with extensive pathway enrichment spanning cell cycle regulation (mitotic cell cycle process, G2/M DNA damage checkpoint, chromosome organization), genomic stability (nuclear DNA replication, APC/C-mediated degradation of cell cycle proteins), and cellular stress responses (response to toxic substance, regulation of membrane potential) (**Supplementary Figure 1**). The convergence of these pathways indicates a multi-target, multi-pathway toxicological mode of action.

### Enhanced expression of key genes in LUAD

**Figure 4:**
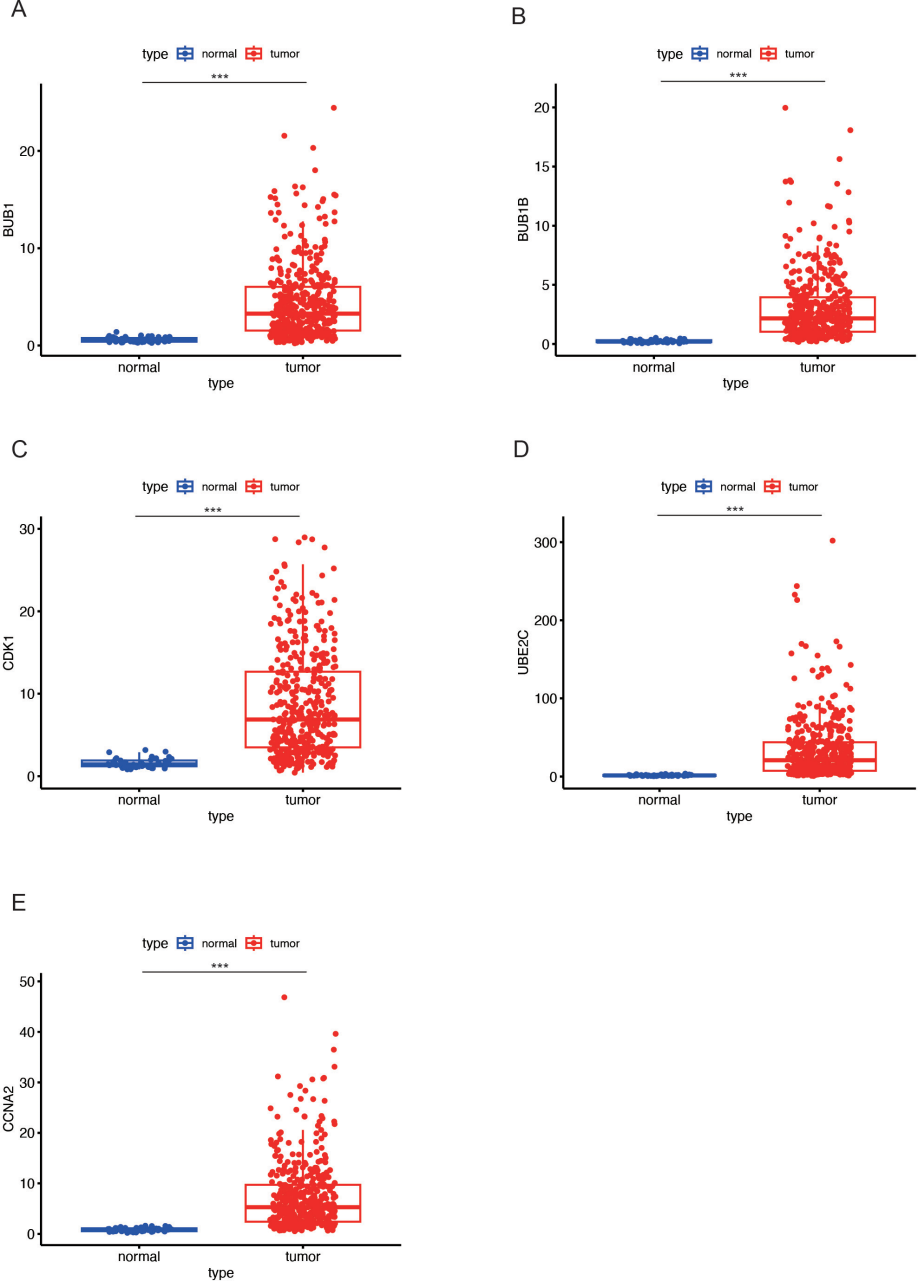
Expression levels of key genes in LUAD tumor and normal tissues. **(A-E)** Box plots showing the differential expression of key genes (BUB1 in **(A)**, BUB1B in **(B)**, CDK1 in **(C)**, UBE2C in **(D)**, and CCNA2 in **(E)** between LUAD tumor tissues (red) and normal tissues (blue). All five genes exhibit higher expression in tumor tissues compared to normal tissues. Data are presented as mean ± SEM. Statistical significance is indicated as *p < 0.05, **p < 0.01, ***p < 0.001.

To further elucidate the expression profiles of the five key markers in LUAD, we examined their expression levels in both normal and tumor tissues. The analysis revealed that the five key genes, including BUB1 (**Figure 4A**), BUB1B (**Figure 4B**), CDK1 (**Figure 4C**), UBE2C (**Figure 4D**), and CCNA2 (**Figure 4E**), exhibited higher expression in LUAD tumor tissues compared to normal tissues in all cases. The marked upregulation of these genes in LUAD indicates their potential involvement in tumorigenesis and progression.

### Molecular docking analysis of BPA and key target proteins in LUAD

**Figure 5:**
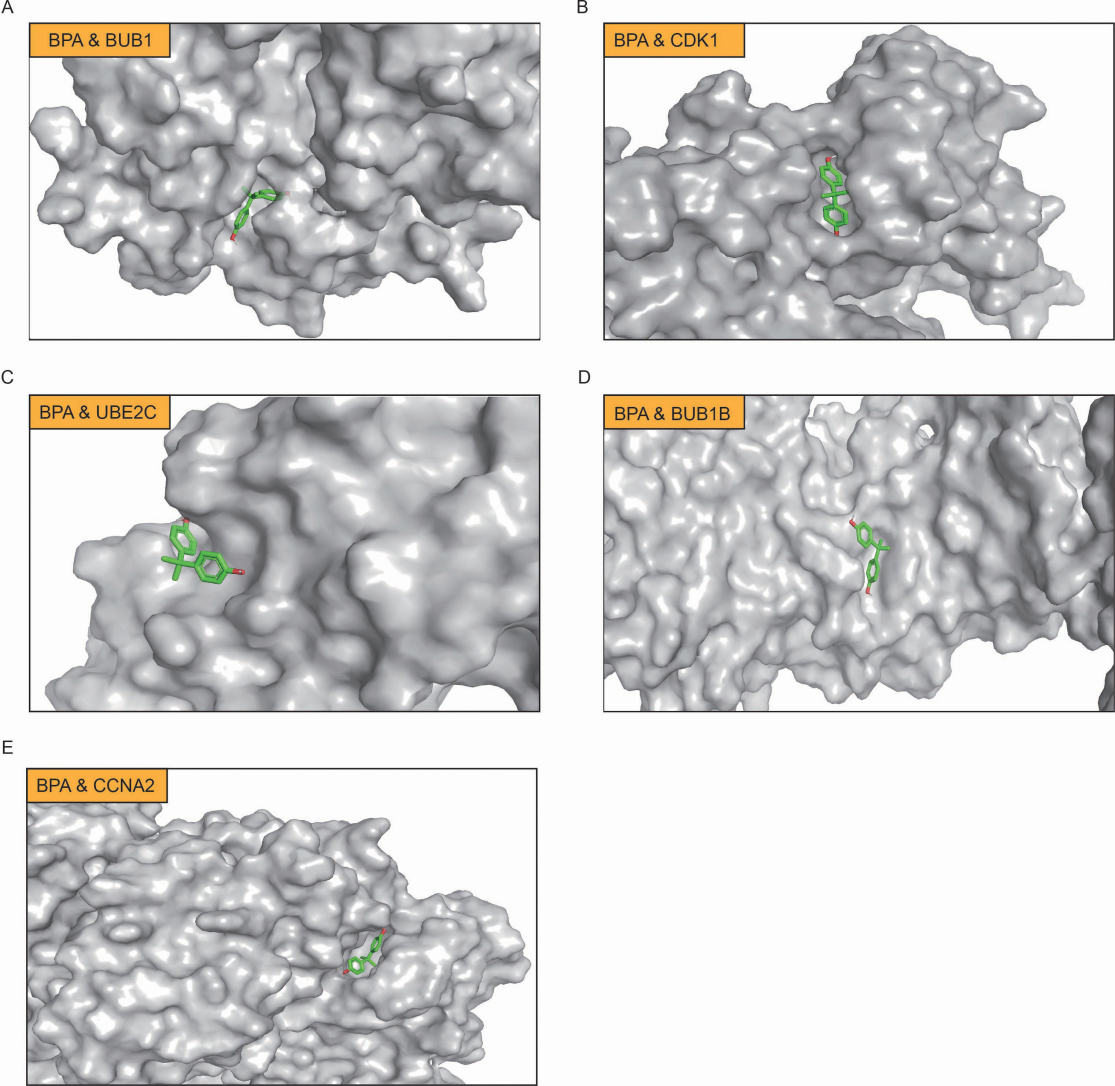
Molecular docking analysis of BPA with key target proteins in LUAD. **(A)** BPA binding to BUB1, **(B)** BPA binding to CDK1, **(C)** BPA binding to UBE2C, **(D)** BPA binding to BUB1B, and **(E)** BPA binding to CCNA2. In each panel, BPA is shown in green, and the binding sites on the respective proteins are highlighted.

Through computational protein docking analysis, we found that BPA interacts with five key proteins (BUB1, CDK1, UBE2C, BUB1B, and CCNA2) at potential binding sites (**Figure 5A-E**; **Supplementary Table S1**). These docking results indicate that BPA can bind to specific sites on these key proteins, potentially interfering with their normal function, which may affect cell cycle regulation and tumorigenesis. Through network toxicology analysis, we further revealed the molecular mechanisms of BPA, indicating that BPA poses a potential risk for lung cancer. Specifically, BPA’s binding to these proteins may influence tumor-related signaling pathways, such as cell proliferation, apoptosis, and DNA repair, thereby promoting the onset and progression of lung cancer.

### High expression of key genes correlates with poor prognosis in LUAD

**Figure 6:**
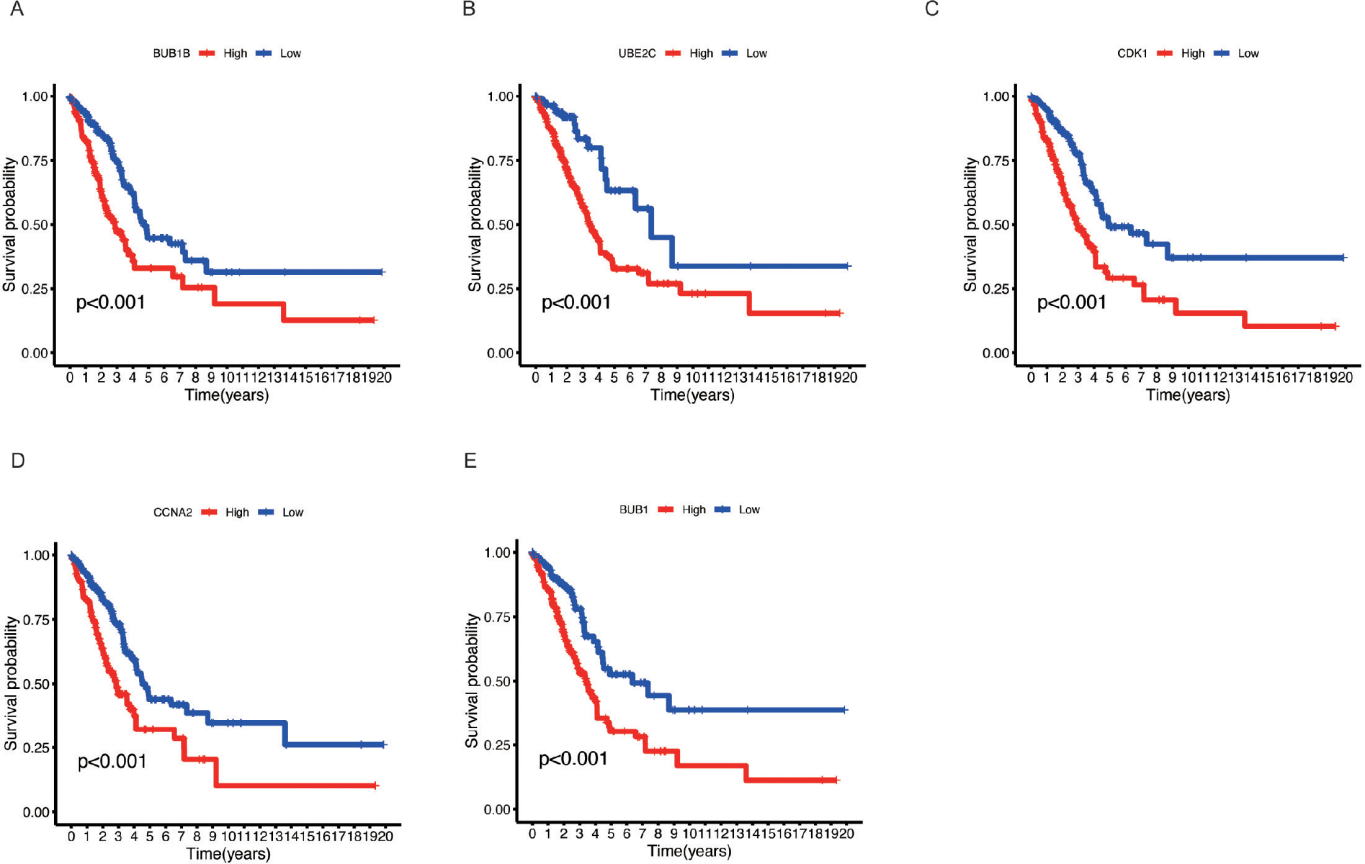
Kaplan-Meier survival analysis of key genes in LUAD. **(A)** BUB1B, **(B)** UBE2C, **(C)** CDK1, **(D)** CCNA2, and **(E)** BUB1. Kaplan-Meier survival curves for LUAD patients based on the expression levels of the five key genes. Patients were divided into high (red) and low (blue) expression groups for each gene.

Survival analysis using Kaplan-Meier curves was performed to assess the prognostic value of five key genes, including BUB1 (**Figure 6A**), UBE2C (**Figure 6B**), CDK1 (**Figure 6C**), CCNA2 (**Figure 6D**), and BUB1B (**Figure 6E**). The analysis revealed that high expression of these genes was associated with poorer survival outcomes in LUAD patients. Patients with high expression of these genes (red curves) exhibited a markedly lower survival probability compared to those with low expression (blue curves), with p-values less than 0.001 in all cases. These results suggest that elevated expression of these genes is linked to increased mortality in LUAD, highlighting as prognostic biomarkers. High expression of these genes may contribute to the aggressive nature of LUAD, underscoring their importance in tumor progression.

### Impact of BPA-related key genes on immune cell infiltration in the tumor microenvironment

**Figure 7:**
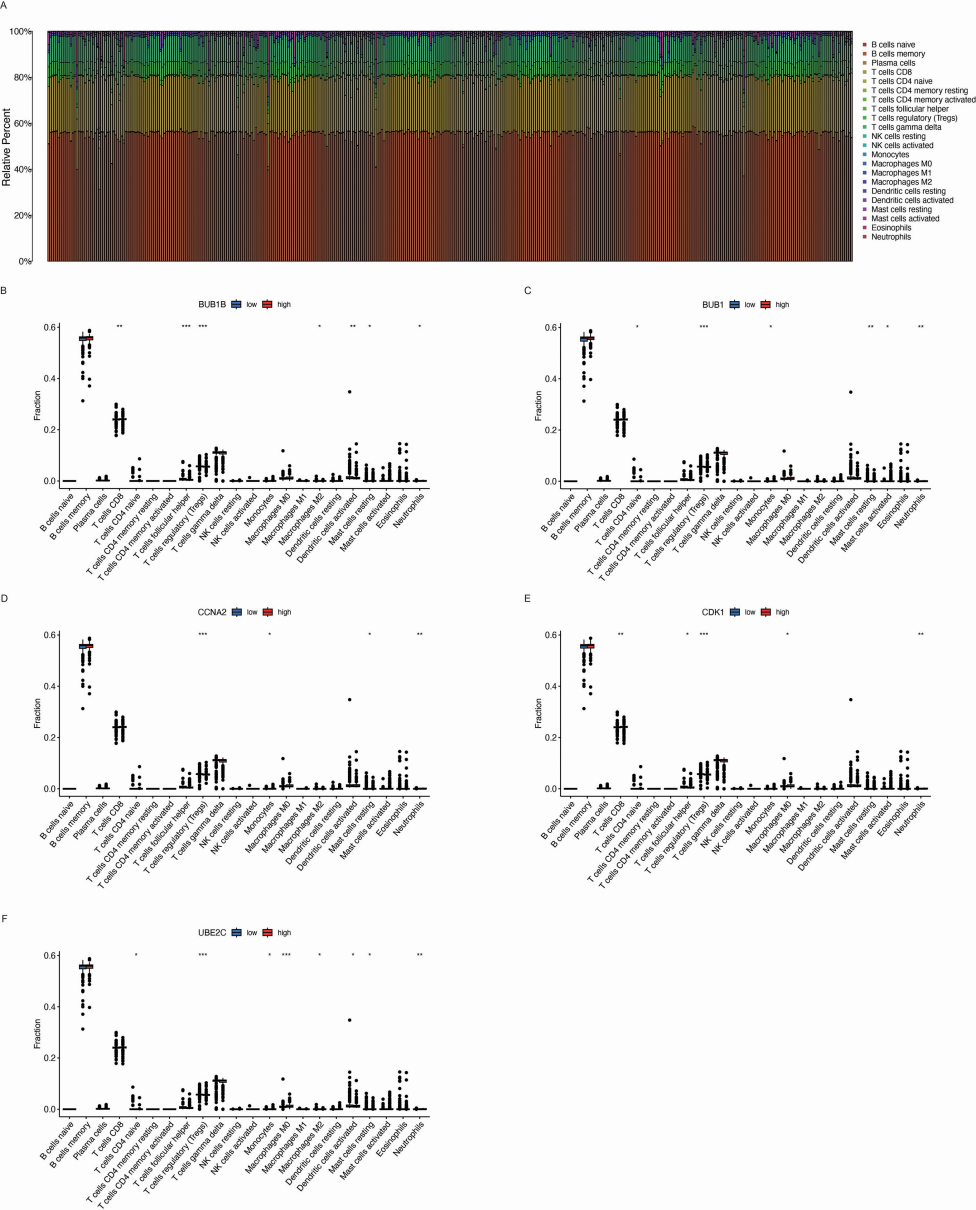
Immune cell infiltration analysis in the tumor microenvironment (TME) of LUAD based on the expression of key genes. **(A)** Relative immune cell infiltration in LUAD samples. The bar chart shows the proportion of different immune cell types within the tumor microenvironment (TME) of LUAD samples, indicating significant variability in immune cell infiltration across different tumor samples. **(B-F)** Box plots depicting the immune cell infiltration levels associated with the expression of five key genes: BUB1B, BUB1, CCNA2, CDK1, and UBE2C. Data are presented as mean ± SEM. Statistical significance is indicated as *p < 0.05, **p < 0.01, ***p < 0.001.

The tumor microenvironment (TME) plays a critical role in the development and progression of lung cancer. We observe that there is significant variability in immune cell infiltration across the different LUAD samples (**Figure 7A**). To investigate whether the key genes associated with BPA influence the TME, we analyzed immune cell infiltration in LUAD samples based on the expression levels of BUB1B (**Figure 7B**), BUB1 (**Figure 7C**), CCNA2 (**Figure 7D**), CDK1 (**Figure 7E**), and UBE2C (**Figure 7F**). The analysis revealed that the high expression of these genes was associated with altered immune cell infiltration patterns. Specifically, high expression of BUB1B, BUB1, CCNA2, CDK1, and UBE2C correlated with an increased presence of T cell subsets, macrophages, and dendritic cells in the TME. These immune cells are key components of the immune response in cancer, and their increased infiltration suggests that the elevated expression of these genes may influence the immune landscape within the tumor. These findings indicate that the high expression of BPA-related key genes could impact immune responses within the TME.

### Correlation between BPA-related key genes and clinical features in LUAD

**Figure 8:**
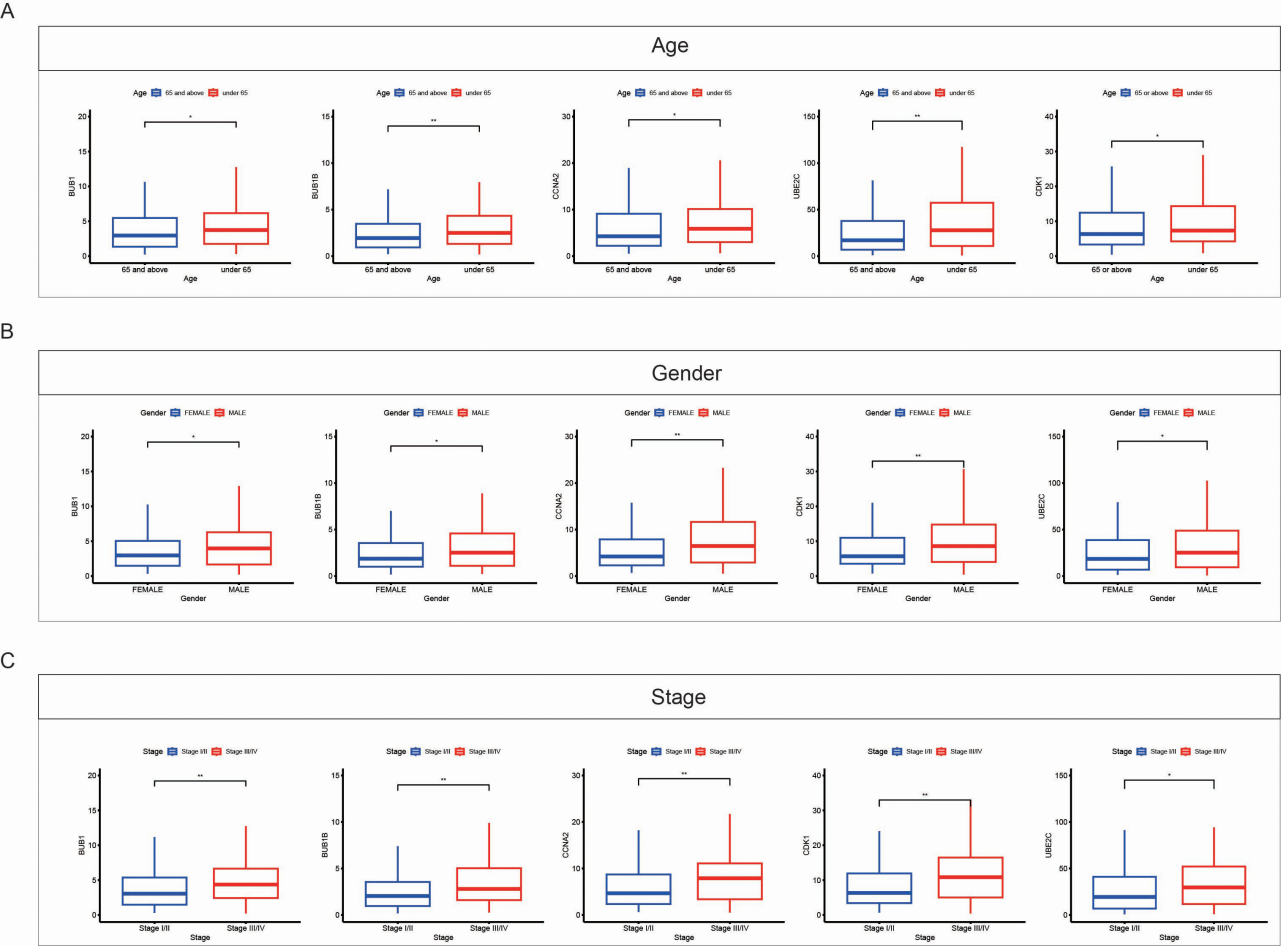
Correlation between expression of key genes and clinical features in LUAD. **(A)** Box plots showing the expression levels of key genes (*BUB1B*, *BUB1*, *CCNA2*, *CDK1*, and *UBE2C*) in LUAD samples based on patient age. Patients aged 65 and above (red) exhibit higher expression of these genes compared to patients under 65 (blue). **(B)** Box plots showing the expression levels of key genes based on gender. Male patients (red) show higher expression of these genes compared to female patients (blue). **(C)** Box plots showing the expression levels of key genes across different cancer stages. Patients with advanced stages (Stage III/IV, red) exhibit higher gene expression compared to those in early stages (Stage I/II, blue). Data are presented as mean ± SEM. Statistical significance is indicated as *p < 0.05, **p < 0.01.

We further analyzed the relationship between the expression levels of the key genes (BUB1B, BUB1, CCNA2, CDK1, and UBE2C) and various clinical factors in LUAD. The analysis was performed based on patient age (**Figure 8A**), gender (**Figure 8B**), and cancer stage (**Figure 8C**). The results revealed significant associations between these genes and clinical variables. Specifically, for patients aged 65 and above, the expression of BUB1B, BUB1, CCNA2, CDK1, and UBE2C was higher compared to those under 65. Additionally, male patients showed higher expression levels of BUB1B, CCNA2, CDK1, and UBE2C than female patients, revealing a gender-related difference in the expression of these genes. In terms of cancer stage, patients with more advanced stages (III/IV) exhibited higher expression levels of these genes compared to patients in earlier stages (I/II), indicating the role of these genes in tumor progression and their association with poor prognosis.

### Single-cell RNA sequencing reveals specific cell-type expression of BPA-related risk genes in LUAD

**Figure 9:**
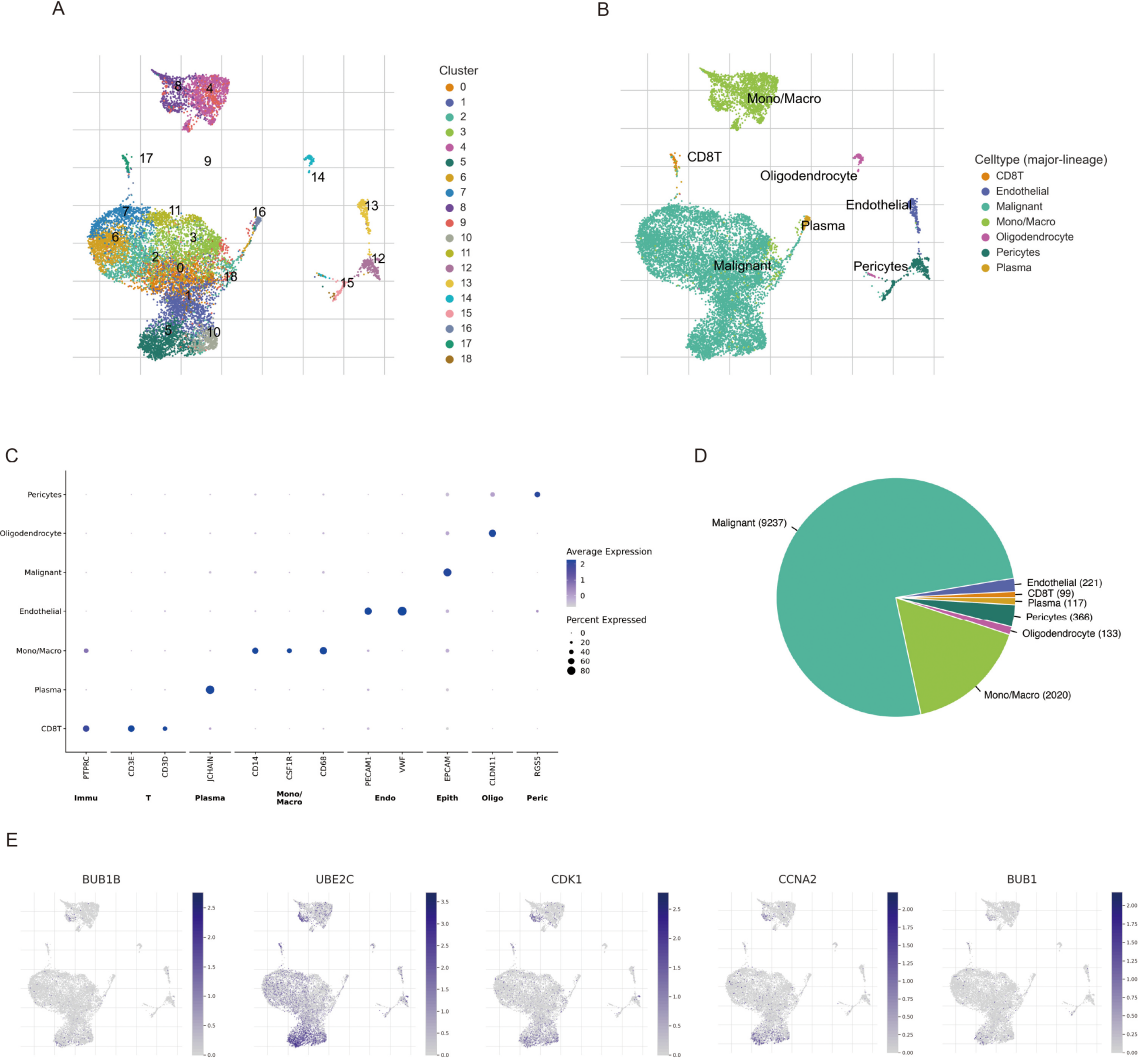
Single-cell RNA sequencing analysis of LUAD samples (GSE143423 dataset) highlighting BPA-related risk gene expression. **(A)** UMAP visualization of cell clusters identified in LUAD tissues. Each color represents a unique cellular cluster. **(B)** Precise annotation of clusters based on known cellular markers, distinguishing epithelial, immune, stromal, and other cell types. **(C)** Heatmap illustrating marker genes specific to each annotated cell cluster, confirming their distinct molecular signatures. **(D)** Bar plot depicting the proportion of identified cell populations within the LUAD tumor microenvironment. **(E)** Expression mapping of BPA-related LUAD risk genes onto UMAP plots, demonstrating gene-specific enrichment particularly within epithelial cells, macrophages, and T cell clusters.

To further delineate the cellular localization and expression patterns of BPA-related risk genes in LUAD, single-cell RNA sequencing data from the GSE143423 dataset were analyzed. UMAP clustering analysis effectively identified distinct cellular subpopulations within LUAD tissues (**Figure 9A**). Based on specific gene expression profiles, these cell clusters were annotated into precise cell types, including epithelial cells, immune cells, stromal cells, and other cellular subtypes (**Figure 9B**). Marker genes representative of each identified cluster demonstrated unique expression patterns, confirming the accuracy and biological significance of the defined subpopulations (**Figure 9C**). The relative proportion of each cellular subgroup revealed variability in their abundance within LUAD samples, highlighting the complexity and heterogeneity of the tumor microenvironment (**Figure 9D**). Importantly, analysis of BPA-associated LUAD risk genes indicated their predominant localization and expression specifically in epithelial cells and certain immune subpopulations, such as macrophages and T cells, supporting possible mechanisms by which BPA may influence tumor progression through modulation of these particular cell populations in the lung cancer microenvironment (**Figure 9E**).

### Toxic effects of BPA on bronchial epithelial cells

**Figure 10:**
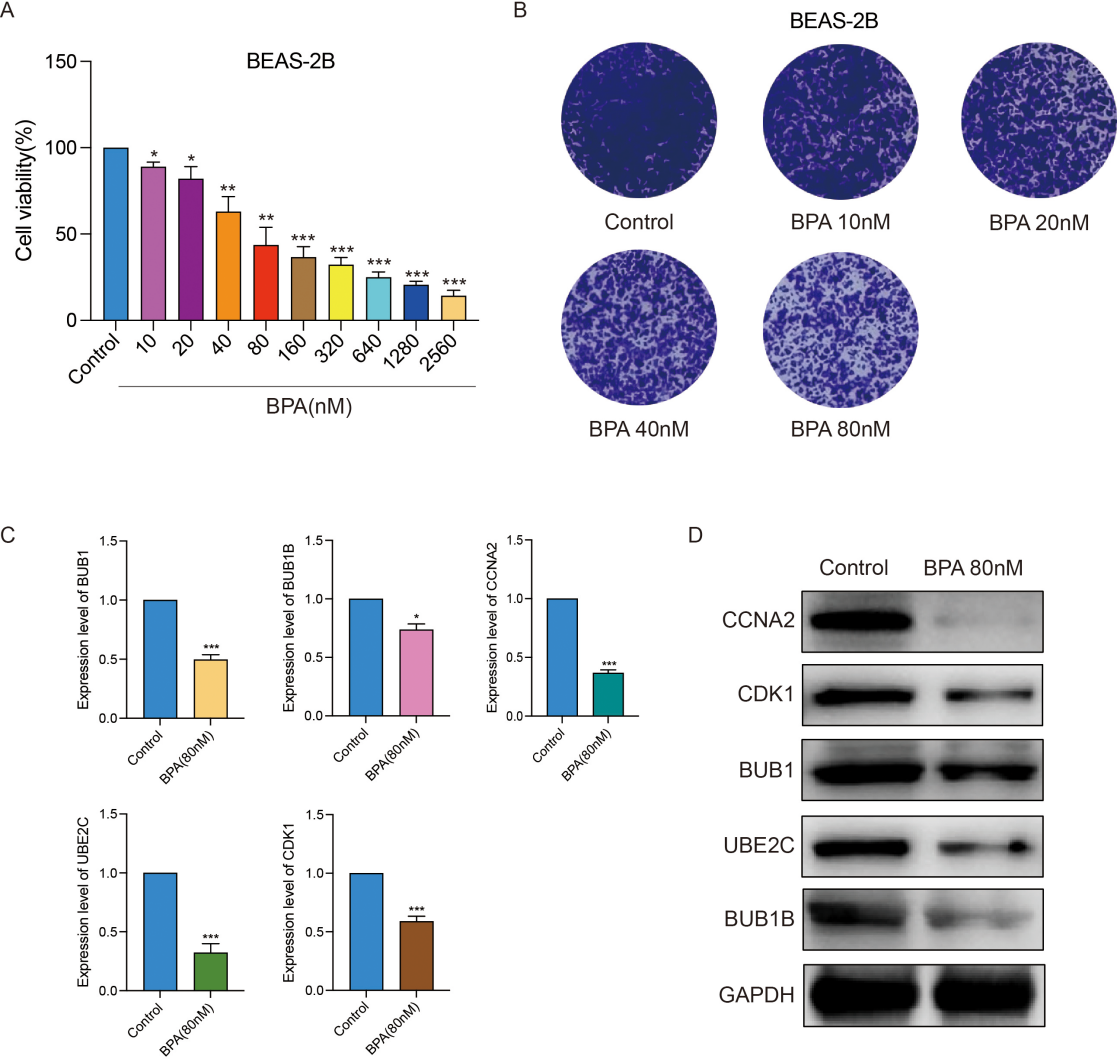
BPA exposure induces cytotoxicity and downregulates key LUAD risk genes in immortalized bronchial epithelial cells (BEAS-2B). **(A)** Cell viability analysis showing dose-dependent cytotoxic effects of BPA on BEAS-2B cells. **(B)** Morphological changes in BEAS-2B cells following BPA treatment at indicated concentrations. **(C)** Western blot analysis displaying significant reductions in protein levels of BUB1, BUB1B, CCNA2, UBE2C, and CDK1 upon BPA exposure (80 nM). GAPDH serves as loading control. **(D)** Quantitative analysis confirming the decreased expression levels of BUB1, BUB1B, CCNA2, UBE2C, and CDK1 after BPA treatment (80 nM), represented as mean ± SEM (**p < 0.01, ***p < 0.001 *vs*. control). Data are presented as mean ± SEM. Statistical significance is indicated as *p < 0.05, **p < 0.01, ***p < 0.001.

To validate the direct effects of BPA on bronchial epithelial cells, immortalized BEAS-2B cells were treated with varying concentrations of BPA. Cell viability assays indicated a dose-dependent cytotoxic effect of BPA on BEAS-2B cells, with significant reductions observed starting at low nanomolar concentrations (**Figure 10A**). Morphological changes consistent with cellular stress were evident with increasing BPA concentrations (**Figure 10B**). Transcriptomic and protein expression analyses demonstrated a marked reduction in the expression of key risk genes BUB1, BUB1B, CCNA2, UBE2C, and CDK1 at both RNA and protein levels following BPA exposure (**Figures 10C, D**). These results underscore the cytotoxic effects of BPA on bronchial epithelial cells.

## Discussion

This study explores the relationship between BPA and LUAD by integrating network toxicology, molecular docking, and clinical data analysis. Previous research has largely focused on BPA’s roles in breast and prostate cancers, it has been shown to activate estrogen receptor signaling, promote oxidative stress, and alter epigenetic regulation (17–19). In contrast, the impact of BPA on lung cancer has received limited attention. Our findings extend this knowledge by identifying LUAD-specific candidate genes (BUB1, BUB1B, CCNA2, CDK1, UBE2C) that may mediate BPA’s carcinogenic effects.

BPA is widely recognized for its endocrine-disrupting properties, which have been linked to various diseases, including cancers such as breast and prostate cancer. However, the specific mechanisms by which BPA contributes to lung diseases, particularly lung cancer, remain poorly understood (20). While earlier studies have emphasized BPA’s systemic endocrine-disrupting activity, our results suggest that its oncogenic influence in LUAD may operate through disruption of cell cycle checkpoints and genomic stability (21–23). Molecular docking analysis of BPA with key target proteins, including BUB1, CCNA2, BUB1B, CDK1, and UBE2C, revealed strong binding interactions, suggesting that BPA may directly interfere with the normal functioning of these proteins. For instance, CDK1 and BUB1 have been associated with aggressive behavior in cancers (24–26), but their direct involvement in environmental chemical–induced lung carcinogenesis had not been demonstrated. By showing strong predicted binding between BPA and these proteins, our study provides a mechanistic bridge linking environmental exposure to dysregulation of core mitotic regulators in LUAD.

Furthermore, previous reports indicated that BPA can modulate immune responses, such as altering T cell activation or macrophage polarization (27, 28). In this study observed that BPA-associated key genes were correlated with altered immune infiltration patterns in LUAD, suggesting that BPA exposure may not only affect tumor cells intrinsically but also remodel the tumor microenvironment. This dual action distinguishes our findings from prior cancer models, where immune modulation was less clearly connected to specific genetic drivers.

Although our findings from gene expression profiling, immune infiltration analysis, and molecular docking provide valuable insights into the potential roles of BPA-associated targets in LUAD, these results are primarily correlative. Further functional assays, such as gene knockdown/overexpression experiments and pathway validation studies, are required to confirm the mechanistic roles of the identified targets.

This study provides valuable insights into the potential risks associated with BPA exposure and its role in LUAD. By combining network toxicology, molecular docking, and clinical data analysis, we identified key genes and pathways involved in BPA-induced LUAD. BPA seems to influence the onset and progression of LUAD by affecting oncogenic genes and immune function. Specifically, the high expression of key genes associated with BPA exposure, such as BUB1, BUB1B, CCNA2, CDK1, and UBE2C, is linked to poor survival outcomes in LUAD patients. Future studies are needed to confirm these findings and explore the detailed mechanisms through which BPA contributes to lung cancer development, ultimately informing risk assessments and public health strategies to minimize exposure to environmental toxins.

## Data Availability

The datasets presented in this study can be found in online repositories. The names of the repository/repositories and accession number(s) can be found in the article/**Supplementary Material**.
