# Supplementary material for: Exploring the carcinogenic potential of bisphenol A in lung adenocarcinoma: molecular mechanisms, key gene insights, and immune microenvironment impacts
Source: Front Immunol. 2025 Oct 16;16:1647807. doi: 10.3389/fimmu.2025.1647807 (PMC12571864; doi:10.3389/fimmu.2025.1647807)
Supplement: Supplementary file 1 [file DataSheet1.docx]

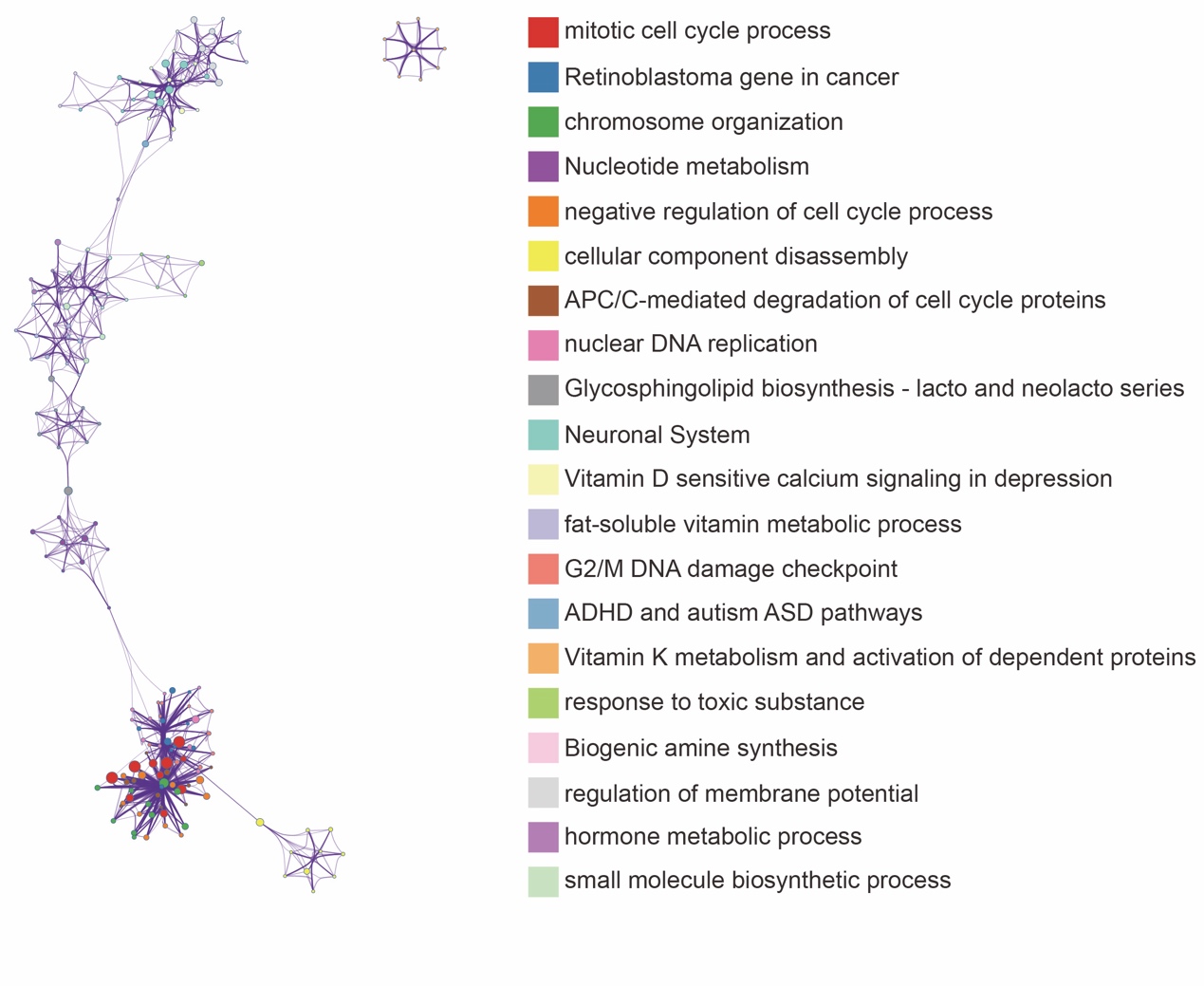


Supplementary Figure 1. Network visualization of enriched pathways identified using the Metascape database.
