# Supplementary material for: Exploring the carcinogenic potential of bisphenol A in lung adenocarcinoma: molecular mechanisms, key gene insights, and immune microenvironment impacts
Source: Front Immunol. 2025 Oct 16;16:1647807. doi: 10.3389/fimmu.2025.1647807 (PMC12571864; doi:10.3389/fimmu.2025.1647807)
Supplement: Supplementary file 2 [file Table1.docx]

| **Table S1.Molecular docking parameters** | | | | | |
| --- | --- | --- | --- | --- | --- |
| Protein | Bingding Energy (kcal/mol) | RMSD(Å) | H-Bonds | Hydrophobic Interaction | Key Residues |
| BUB1 | -7.3 | 0.01 | 2 | 5 | Phe 818, Ser 854, His 856, Val 865, Lys 785 |
| BUB1B | -7.5 | 0 | 0 | 6 | Arg 60, Tyr 64, Arg 67, Phe 410, Glu 418 |
| CCNA2 | -6.3 | 0.04 | 2 | 2 | Val 219, Glu 220, Glu 224, Asn 415 |
| CDK1 | -6.7 | 0.01 | 2 | 8 | Ile 10, Val 18, Ala 31, Phe 80, Ser 84, Asp 86, Lys 89, Leu135, Ala 145 |
| UBE2C | -5.8 | 0.03 | 3 | 2 | Asp 116, Ser 134, Ser 137, Leu 138, Asn 143 |
